# Gut Dysbiosis Serine–Glycine Metabolism and Glioblastoma: Exploring Therapeutic Opportunities

**DOI:** 10.3390/cancers18111717

**Published:** 2026-05-25

**Authors:** Micol Mangano, Maria Cristina Ermio, Fabio Sciubba, Michele De Rosa, Giuseppina D’A lessandro, Cristina Limatola, Maria Rosito

**Affiliations:** 1 Department of Physiology and Pharmacology, Sapienza University, 00185 Rome, Italy; micol.mangano@uniroma1.it (M.M.); ermio.1886635@studenti.uniroma1.it (M.C.E.); 2 Department of Environmental Biology, Sapienza University, 00185 Rome, Italy; fabio.sciubba@uniroma1.it; 3 NMR-Based Metabolomics Laboratory (NMLab), Sapienza University, 00185 Rome, Italy; michele.derosa@uniroma1.it; 4 Interdepartmental Center of Applied Sciences for the Protection of the Environment and Cultural Heritage (CIABC), Sapienza University, 00185 Rome, Italy; 5 IRCCS Neuromed, 86077 Pozzilli, Italy; giuseppina.dalessandro@uniroma1.it (G.D.l.); cristina.limatola@uniroma1.it (C.L.); 6 Department of Physiology and Pharmacology, Sapienza University, Laboratory Affiliated to Institute Pasteur Italia, 00185 Rome, Italy; 7 Department of Life Sciences, Health and Health Professions, Link Campus University, 00165 Rome, Italy; 8 Center for Life Nanoscience & Neuroscience, Istituto Italiano di Tecnologia@Sapienza, 00161 Rome, Italy

**Keywords:** gut–brain axis, serine–glycine metabolism, gut microbiota, glioblastoma (GBM), metabolic reprogramming

## Abstract

The gut–brain axis is a central regulatory network linking dietary habits, metabolic homeostasis, and brain function through bidirectional communication among the intestine, microbiota, and central nervous system. Acting as a key mediator, the gut microbiota translates environmental and nutritional factors into systemic outcomes that influence both health and disease. Within this context, serine and glycine metabolism emerges as a critical yet underexplored hub connecting microbial activity with brain regulation. Changes in gut microbial composition can profoundly affect host one-carbon metabolism and amino acid availability, shaping systemic physiology and neural processes. In this review, we outline a biochemical framework in which gut microbiota alterations influence brain and liver serine/glycine (ser/gly) metabolism, driving the hepatic production of secondary metabolites, including taurine-conjugated bile acids. We delineate how gut–brain axis pathways orchestrate systemic and neural functions, and highlight glioblastoma (GBM) as a pathological context where hijacked serine–glycine metabolism fuels tumor growth, stemness, and therapy resistance. By focusing on the interplay between gut microbiota, ser/gly metabolism, and brain tumor biology, this review offers a cohesive perspective on translational interventions. Glycine-centered pathways emerge as promising targets to modulate the gut–brain–tumor axis, opening new avenues to influence GBM progression and enhance therapeutic strategies.

## Simple Summary

This review synthesizes current evidence on the interplay between gut dysbiosis and serine/glycine metabolism in glioblastoma, integrating signals across the gut, liver, and brain to define a coherent multi-organ metabolic framework and identify actionable therapeutic targets. It outlines a forward-looking roadmap for research aimed at moving beyond single-organ perspectives toward systems-level interventions that combine metabolic modulation with microbiota-based strategies in GBM. Growing evidence indicates that gut microbiota dysbiosis reshapes serine/glycine metabolism along the gut–brain axis, with downstream effects on one-carbon metabolism and systemic amino acid availability that may support tumor-favoring conditions. In this context, glycine accumulation is increasingly recognized as a driver of glioblastoma growth, stemness, and therapy resistance, positioning serine/glycine metabolic rewiring as a central vulnerability and a promising target for integrated therapeutic approaches.

## 1. The Gut–Brain Axis: An Integrated and Hierarchically Organized Anatomical System

Over the past decade, scientific research has increasingly shown that gut microbiota is a vital determinant of human health. It participates actively in nutrient metabolism, assisting in the breakdown of complex dietary components that humans alone cannot digest. These metabolic activities produce key compounds such as short-chain fatty acids, which support intestinal barrier integrity, influence energy balance, and regulate immune responses [1].

As a result, gut microbiota research has become a central topic in modern biomedical science, attracting interest not only in nutrition and gastroenterology but also in immunology, neuroscience, and personalized medicine [2]. This highlights the bidirectional communication between the gastrointestinal tract and the central nervous system (CNS), the gut–brain axis, suggesting that gut microbes can influence mood, cognition, and neural signaling.

The gut–brain axis is organized as a hierarchical and integrative network of anatomical structures. At the top of this hierarchy lies the CNS, particularly the brainstem, hypothalamus, limbic system, and cortical areas, which function as the main integrative centers [3]. These regions receive sensory information from the gut, modulating motility, secretion, vascular tone, and immune activity, especially during stress or emotional states [4,5,6,7].

At the peripheral level, the enteric nervous system (ENS) forms an extensive and semi-autonomous neural network embedded within the gut wall [8]. The ENS operates as a critical intermediate node in the hierarchy, integrating local sensory inputs from the intestinal lumen and coordinating motor and secretory reflexes, while maintaining constant communication with the CNS via autonomic pathways. Closely associated with the ENS is the intestinal epithelium, which serves as both a physical barrier and a sensory interface. Specialized epithelial cells, enteroendocrine cells, detect nutrients and microbial metabolites and translate these signals into hormonal and neuroactive outputs that act locally on enteric neurons or systemically on the brain [9].

**Figure 1:**
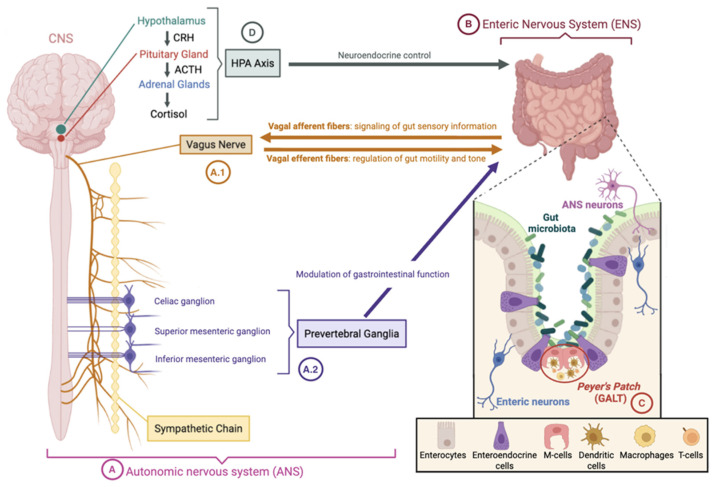
**Structural and Functional Architecture of the Bidirectional Gut–Brain Axis.** (**A**) Autonomic Pathways. Extrinsic neural regulation is divided into two primary branches: A.1. Parasympathetic Pathway: mediated primarily by the vagus nerve, this branch provides bidirectional signaling. Efferent (motor) fibers travel from the hindbrain to synapse within the gut wall, while afferent (sensory) fibers transmit stimuli from the gastrointestinal tract back to the CNS. A.2. Sympathetic Pathway: originating from the spinal cord, preganglionic fibers pass through the sympathetic chain to form splanchnic nerves. These nerves synapse within prevertebral (visceral) ganglia (celiac, superior mesenteric, and inferior mesenteric), from which postganglionic fibers extend to the gut to modulate blood flow, immune activation, and epithelial permeability, particularly during the stress response. (**B**) The Enteric Nervous System (ENS). Operating as a semi-autonomous intermediate node embedded within the layers of the gastrointestinal (GI) wall, the ENS integrates extrinsic autonomic signals (from A.1 and A.2) with local luminal stimuli to coordinate motor and secretory reflexes, while maintaining a constant relay with the CNS. (**C**) The Mucosal Interface and GALT. The intestinal epithelium serves as a sensory-motor interface where specialized enteroendocrine cells and M cells sample microbial metabolites and antigens within Peyer’s patches (GALT). These signals are transduced into hormonal and neural outputs that coordinate mucosal immune defense and systemic signaling. (**D**) Neuroendocrine Signaling. The Hypothalamic–Pituitary–Adrenal (HPA) axis complements these neural pathways, providing a systemic endocrine route for brain-to-gut communication via the corticotropin-releasing hormone (CRH) and adrenocorticotropic hormone (ACTH) -mediated release of cortisol and other stress-related hormones.

Within this anatomical framework, the gut-associated lymphoid tissue (GALT) links immune surveillance to neural signaling [10]. Immune cells within the gut mucosa release cytokines and inflammatory mediators that can influence enteric neurons, vagal afferents, and ultimately CNS activity. Finally, the hypothalamic–pituitary–adrenal (HPA) axis represents a higher-order neuroendocrine pathway within the hierarchy, connecting stress-related brain regions to gut physiology through hormonal signaling [11,12]. Together, these structures and pathways form a multi-layered network in which information flows from the gut to the brain and back again, allowing the gut–brain axis to coordinate physiological homeostasis, emotional regulation, and adaptive responses to internal and external stimuli (Figure 1).

## 2. Gut Microbiota Variability and Dysbiosis: Environmental Drivers and Experimental Murine Models

Dysbiosis refers to an alteration of the microbial ecosystem characterized by the loss and/or expansion of specific bacterial taxa, resulting in reduced diversity and an imbalance in microbiota composition. The human gut microbiota displays remarkable taxonomic complexity; according to the Weizmann Institute of Science reference dataset, western and non-western populations collectively harbor approximately 3594 microbial species, many of which had not been previously characterized [13]. Although earlier research sought to define a single, universal gut microbiota profile representative of healthy individuals, this concept is now considered overly simplistic. Instead, accumulating metagenomic evidence indicates substantial interindividual variability, while revealing the presence of conserved functional “core” metabolic pathways that are consistently shared across human populations [14]. It has been reported that the human minimal gut metagenome “includes functions known to be important to the host–bacterial interaction, such as degradation of complex polysaccharides, synthesis of short-chain fatty acids, indispensable amino acids, and vitamins [15].

The gut microbiome has been proposed as a biological mediator linking socioeconomic status to health outcomes. Evidence indicates that social and environmental determinants exert a stronger influence on microbiome composition than host genetics, supporting the concept of a “sociobiome,” whereby individuals sharing similar socioeconomic environments exhibit comparable microbial profiles. These factors—including diet quality, hygiene practices, antibiotic exposure, and living conditions—shape gut microbial communities [16] and are classifiable as (i) host-dependent factors (genetics, general health status, lifestyle); (ii) environmental factors (diet, xenobiotics, hygiene) [17].

While it is now known that the unique gut microbiota stays stable throughout the life of the individual and short-term nutritional changes are not drastically modifying its composition, daily transient fluctuations are observed, with diet playing an important role as a microbial influencing factor [18,19].

It has been reported that high-fat content and high-sugar content diets—typically associated with western diets—are responsible for a higher relative abundance of the Firmicutes in the mouse distal gut, which positively correlate with obesity [20]. In 2014, Schultz et al. proved that intestinal carcinogenesis in K-rasG12Dint mice fed with a high-fat diet “may be based on marked shifts in bacterial communities rather than on the development of obesity and metabolic disorder” [21].

Although many lifestyle factors influencing the microbiome have been identified, significant knowledge gaps remain. One of the most controversial aspects in obesity-related microbiota research is the Firmicutes/Bacteroidetes ratio. Contradictory results have been observed when comparing the microbiota of normal-weight and obese subjects, making it difficult to associate variations in the Firmicutes/Bacteroidetes ratio with a specific health status [22].

Current microbiome research is largely based on populations from socioeconomically developed countries, potentially biasing interpretations of microbiome variation and its links to health and disease. In addition, the under-representation of minority groups limits our understanding of how context, history, and dynamic changes in the microbiome influence disease risk. Recent advances highlight ageing and ethnicity as key contributors to microbiome variability, offering important insights for the development of microbiome-based diagnostics and therapeutics [23].

In addition to the growing interest in characterizing the composition and heterogeneity of the human gut microbiota, studies conducted in experimental murine models have played a crucial role over the past decade. These models represent indispensable tools for mechanistic investigations, allowing causal relationships to be established and enabling detailed analysis of the contribution of the gut microbiome to disease onset, progression, and therapeutic response.

### From Germ-Free to Humanized Mice: Experimental Models to Explore the Gut Microbiome and the Gut–Brain Axis

Research using germ-free (GF) mice has provided some of the strongest evidence that the gut microbiota plays a key role in gut–brain communication. These studies directly address whether the microbiota can influence the nervous system and have yielded important insights into its impact on CNS development and function, particularly in relation to neuropsychiatric disorders. Findings show that growing up without microorganisms leads to significant alterations in behavior and brain function [24,25].

Although GF models have also been developed in other species [26,27], GF mice remain the most widely used and powerful tool. Their main advantage is the ability to introduce defined bacterial strains, including candidate psychobiotics, and study their effects in a gnotobiotic condition [28]. In addition, GF mice can be colonized with human [29,30] or disease-associated microbiota [31], allowing researchers to investigate how specific microbial communities contribute to homeostasis and disease mechanisms.

One of the key pieces of evidence highlighting the importance of the gut microbiota in maintaining CNS cellular homeostasis comes from a study by Erny and colleagues [32], who showed that a complex gut microbiota supports microglial maintenance under steady-state conditions, whereas its absence leads to defects in microglial maturation, differentiation, and function. By regulating microglial innate immune activity, the gut microbiota primes the brain for rapid immune responses to pathogens or danger signals, as evidenced by impaired microglial function in GF and antibiotic-depletion models. The latest represents another strategy used to eliminate the resident gut microbiota and consists of the administration of non-absorbable antibiotics (gentamicin, vancomycin, cefoxitin, metronidazole), which selectively and effectively deplete the intestinal bacterial flora without directly affecting the CNS development and homeostasis [32,33,34].

## 3. Gut Microbiota and Serine/Glycine Metabolism

The gut microbiota acts as a metabolically active organ that produces a wide range of bioactive compounds. These metabolites include protective molecules, such as short-chain fatty acids and secondary bile acids, alongside potentially deleterious products, including precursors of uremic toxins and advanced glycation end products [35,36,37]. Moreover, current evidence on the role of microbial amino acids in regulating host amino acid balance revealed that gut microbiota facilitates metabolite production by utilizing dietary- and host-derived amino acids for protein synthesis and by driving nutrient metabolism through conversion and fermentation [38].

Gut microbes can synthesize several essential amino acids de novo, thereby contributing to host amino acid homeostasis [39]. This contribution is finely tuned by diet, which shapes microbial composition and metabolic capacity, resulting in a dynamic equilibrium closely linked to dietary habits and lifestyle.

The impact of long-term nutritional changes on the gut microbiota has not yet been fully elucidated; however, studies in mice show that metabolic pathways altered by a high-fat diet involve glycine, serine, and threonine metabolism, as well as the biosynthesis and metabolism of several other amino acids [40].

An animal-based diet is able to induce a shift in human microbial metabolism, significantly lowering carbohydrate fermentation and increasing amino acid fermentation [41]; moreover, subjects going on a vegan diet present elevated serum glycine concentration, despite lower dietary glycine intake, suggesting that the vegan-diet-related alteration of gut microbial composition significantly modifies serum and stool metabolomes [42].

### Hepatic Glycine Metabolism During Gut Microbiota Alteration

Liver is a keystone of the gut–brain axis since it is directly involved in processes associated with nutrient adsorption [43] and, given the role of the bile salts in the digestion of fatty acids and cholesterol, it is able to release molecules in the same environment of gut microbiota, thus chemically correlating gut and microbiota. At the same time, the liver is known to largely employ the amino acid glycine, not only to improve the solubility of catabolic end metabolites (i.e., benzoic acid converted to hippuric acid [44]), but also in the synthesis of some bile salts [45,46].

However, little is known about the response of liver metabolism in the presence of an altered microbiota.

It has recently been reported that an alteration of the gut microbiota, mediated by the administration of non-absorbable antibiotics, leads to an increase in glycine derivatives in mouse fecal water [34]. Within this experimental approach, the fate of fully ^13^C-labeled glycine administered orally to mice was analyzed through ^13^C nuclear magnetic resonance (NMR) spectroscopy, which allows the identification and quantification of the different isotopomers of the same metabolite in control and in chronic antibiotic-treated mice [47,48].

**Figure 2:**
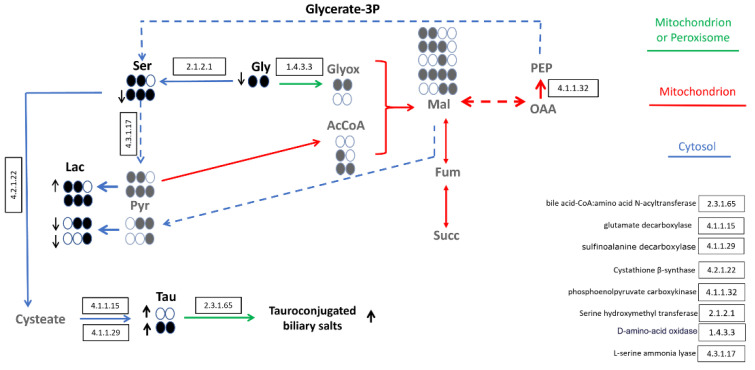
**Schematic representation of the metabolic fate of fully ^13^C-labeled glycine in liver metabolism.** The network summarizes the main hepatic pathways involving glycine, including its interconversion with serine, conversion to pyruvate and lactate, entry into central carbon metabolism via acetyl-CoA, and its contribution to tricarboxylic acid (TCA) cycle intermediates (succinate, fumarate, malate, and oxaloacetate), as well as phosphoenolpyruvate (PEP) formation. Glycine metabolism through glyoxylate and its links with mitochondrial and peroxisomal reactions are also indicated. In addition, glycine-derived cysteate and taurine formation, leading to tauroconjugated bile salts, are shown as part of sulfur amino acid metabolism. Metabolites are depicted as clusters of circles in equal numbers to the carbon atoms present in the molecular backbone. White circles indicate unlabeled ^12^C atoms while black circles indicate ^13^C-labeled atoms. Dark-colored metabolites represent compounds directly detectable by NMR, whereas Gray-colored metabolites are not directly observable. Upward and downward black arrows indicate relative increases or decreases in metabolite abundance after antibiotic administration. Solid arrows denote single, direct enzymatic reactions, while dashed arrows represent multi-step processes or indirect pathways. Arrow colors indicate subcellular compartmentalization, as specified in the legend. Enzymes catalyzing each conversion are indicated by their KEGG Enzyme Commission (EC) numbers and listed in the bottom-right corner.

Isotopomers differ according to the position and number of labels in the carbon skeleton, so considering the carbon isotopes ^12^C and ^13^C in the carbon skeleton of a molecule, an isotopomer is one of the possible ^13^C labeling states in which that molecule is found [48]. Given the current knowledge of liver biochemical pathways [49,50], it was possible to summarize the metabolic fate of fully labeled Gly in the mouse liver in Figure 2, thereby identifying the labeled isotopomers of lactate (Lac), glycine (Gly), taurine (Tau), and serine (Ser).

The identified reactions occur in several hepatic cell organelles, like peroxisomes and mitochondria, and reflect elevated tauroconjugates and bile salts in fecal water [34]. This evidence supports the notion of a bidirectional crosstalk between the gut microbiota and the liver, whereby microbiota alterations influence hepatic glycine metabolism within the coordinated regulation of bile salt and tauroconjugate production [51].

## 4. Gut Microbiota and Serine/Glycine Metabolism in Pathological Conditions: Focus on Glioblastoma

Changes in glycine metabolism were also observed in many gut microbial alterations caused by different diseases, such as cancer [34,52,53,54], chronic kidney disease [55], and obesity and metabolic disorders [56,57].

Commonly observed in Western high-fat diets, reduced levels of Bacteroidetes-derived fecal glycine are associated with increased inflammation and may contribute to the progression of atherosclerosis and liver injury in the Low-Density Lipoprotein Receptor Ldlr-/- mouse model [58]. In line with this evidence, dietary glycine supplementation has been shown to reshape gut microbial composition and to enhance intestinal barrier function in weaned piglets. These effects include reduced circulating levels of IL-1α, IL-1β, TNF-α, and CCL2, as well as preservation of the tight-junction proteins Claudin-1, Claudin-4, and ZO-1, which are otherwise downregulated by high-fat diets [59].

It has been suggested that the intestinal microbiota associated with colitis can regulate glycinergic neurotransmission, thereby influencing anxiety-related and social behaviors in mice [60].

Disruptions in gut microbial balance can result from various factors, including dietary imbalances, infections, environmental stressors, and pharmacological interventions, ultimately affecting microbial diversity and predisposing the host to neurological diseases [61,62,63].

Recently, many studies have implied correlations between gut microbiota and brain pathologies, such as mild cognitive impairment [64], Parkinson’s disease [65,66], Alzheimer’s disease [67,68], schizophrenia [69,70], epilepsy [71,72], autism spectrum disorder [73,74], traumatic brain injury [75,76], and brain cancer [33,34,77].

Currently, understanding the underlying mechanism that links gut microbiota modulation to the progression of glioblastoma (GBM) is a trending topic, as it represents a possible tool to counteract the tumor.

GBM is the most common and aggressive primary malignant brain tumor, with incidence increasing with age and peaking in the elderly population [78,79,80]. Recent updates to the World Health Organization classification have defined GBM as an isocitrate dehydrogenase (IDH) wild-type, grade 4 glioma [81].

Despite its lower incidence compared to more common cancers, GBM poses a disproportionate clinical burden due to its aggressiveness, therapeutic resistance, and poor long-term survival [79,82,83].

Although most GBMs occur sporadically, some epidemiological studies have associated both environmental and genetic factors with disease risk [84,85,86,87,88,89,90,91,92].

New evidence supports the hypothesis that GBM patients present profound alterations of gut microbiota compared to healthy subjects. As analyzed by 16S rRNA gene amplicon sequencing, the structure of the gut microbial community reveals higher abundances of Proteobacteria, Fusobacteria, and Bacteroidetes, and reduced abundances of Firmicutes, Actinobacteria, and Verrucomicrobia [93,94].

Upon antibiotic (ABX) administration in a syngeneic mouse model of glioma, it has been documented that the reduction of microbial signals from Prevotellaceae, Rikenellaceae, and Helicobacteraceae or the increased signals from Burkholderiales may modulate immunosurveillance in the brain, boosting a pro-tumoral microenvironment [33].

Recent advances in unraveling the mechanism of the observed pro-tumoral effect of dysbiosis have proposed glycine as a metabolite that may guide the microenvironment towards a tumor-supporting phenotype in glioma-bearing mice with ABX-altered gut microbiota [34]. By employing proton nuclear magnetic resonance (^1^H NMR) spectroscopy, Tiwari et al. quantified glycine concentrations across different brain regions under both physiological and pathological conditions, including GBM. Physiological glycine concentrations are typically in the submicromolar range, averaging approximately 0.3 µM in plasma and 0.05 µM in cerebrospinal fluid (CSF). In contrast, GBM is associated with a marked elevation in glycine levels, which can reach millimolar concentrations. Within the lesion area, glycine levels may range from approximately 3 mM up to 7.5 mM, indicating a substantial increase compared to physiological conditions [95]. These findings further highlight the critical role of glycine in GBM pathophysiology.

## 5. Metabolic Rewiring in GBM: The Central Role of Ser/Gly Pathways

Like many other cancers, GBM exhibits a distinctive metabolic profile characterized by the Warburg effect, in which tumor cells rely on glycolysis for ATP production, even in the presence of oxygen [96].

**Figure 3:**
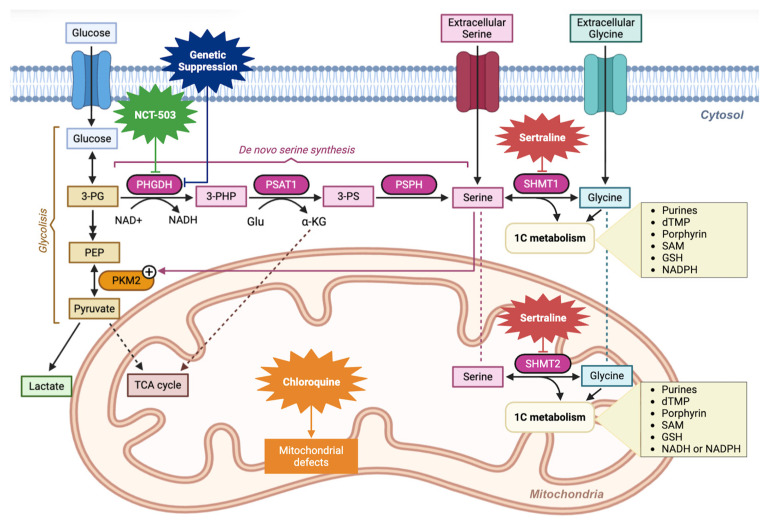
**Metabolic Reprogramming in GBM and Therapeutic Targeting of the Ser/Gly Biosynthetic Pathway.** Driven by the Warburg effect, GBM cells exhibit profound metabolic plasticity characterized by accelerated aerobic glycolysis (enzymatic steps are indicated in black arrows and pathways intermediates in orange boxes) A major biosynthetic branch of this glycolytic flux involves the diversion of 3-phosphoglycerate (3-PG) into the de novo serine synthesis pathway (enzymes are represented in dark pink circles, and metabolic intermediates are enclosed in light pink boxes). This process is initiated by phosphoglycerate dehydrogenase (PHGDH), which oxidizes 3-PG to 3-phosphohydroxypyruvate (3-PHP) with the concomitant reduction of NAD^+^ to NADH. Subsequently, phosphoserine aminotransferase 1 (PSAT1) catalyzes the transamination of 3-PHP, utilizing glutamate as an amino donor to yield 3-phosphoserine (3-PS) and α-ketoglutarate (α-KG), which subsequently enters the Tricarboxylic Acid Cycle (TCA cycle) (red box). The pathway culminates with the dephosphorylation of 3-PS by phosphoserine phosphatase (PSPH), the rate-limiting step, to produce serine. Intracellular serine is partitioned between the cytosol and mitochondria (represented by the pink dotted line), where cytosolic SHMT1 and mitochondrial SHMT2 (enzymes are enclosed in dark pink circles) mediate the reversible interconversion of serine and glycine. This enzymatic axis supplies hydroxymethyl groups, which are essential to sustain one-carbon (1C) metabolic flux, (represented in yellow) which provides fundamental precursors for de novo purine and thymidylate (dTMP) synthesis, supports mitochondrial redox homeostasis by regenerating NADH and NADPH, and sustains S-adenosylmethionine (SAM) production. Glycine further supports the 1C pool and acts as a fundamental precursor for porphyrin, glutathione (GSH), and purine synthesis. Notably, serine acts as an allosteric activator of Pyruvate Kinase M2 (PKM2), sustaining the aerobic glycolytic flux through the production of pyruvate (indicated by the pink arrow). Depending on the cell metabolic needs, pyruvate can be either reduced to lactate or diverted towards the TCA cycle. In GBM, the significant upregulation of the ser/gly metabolic pathways has been exploited as a potential therapeutic target. As illustrated, Sanchez et al. tested Sertraline and Chloroquine joint activity. Sertraline (red) is a common antidepressant and acts as a dual SHMT1/2 inhibitor, effectively impairing ser/gly conversion and consequently disrupting biosynthetic and redox functions across both the cytosolic and mitochondrial compartments. When combined with Chloroquine (orange), which compromises mitochondrial structural integrity, a synergistic reduction in cell survival is observed. Furthermore, Liu et al. exploited PHGDH upregulation in GBM and GSCs, targeting the enzyme through a combination of pharmacological inhibition, using NCT-503 (green), and genetic ablation techniques (blue), effectively impairing serine biosynthetic flux.

Beyond energy generation, glycolysis provides critical anabolic precursors that support rapid proliferation, with a central role played by the de novo serine biosynthesis pathway. In this pathway, the glycolytic intermediate 3-phosphoglycerate (3-PG) is diverted through a three-step enzymatic cascade involving phosphoglycerate dehydrogenase (PHGDH), phosphoserine aminotransferase 1 (PSAT1), and phosphoserine phosphatase (PSPH), which catalyzes the final conversion to serine (Figure 3) [97].

Serine is a fundamental substrate for diverse biosynthetic and signaling pathways, acting as a primary donor of one-carbon units to the folate cycle. This integration occurs via one-carbon metabolism, a biochemical network encompassing the folate and methionine cycles, to produce essential building blocks for cellular function and proliferation [95]. Central to this axis is the enzymatic conversion of serine to glycine by serine hydroxymethyltransferase (SHMT1 in the cytosol and SHMT2 in mitochondria), a process that transfers a hydroxymethyl group to the folate pool, fueling one-carbon metabolism [98].

The ser/gly pathway is frequently upregulated in cancers, with overexpression observed in approximately 30% of tumors to sustain metabolic flux through both endogenous synthesis and extracellular uptake [99,100,101,102]. In ser/gly-dependent GBM cells specifically, this metabolic shift is characterized by the extensive rerouting of glucose-derived carbon from glycolysis into these biosynthetic pathways, which are essential to support the high biosynthetic demands of rapid cellular proliferation [99].

Through its interface with one-carbon metabolism, the folate cycle is crucial for the de novo synthesis of adenosine, guanosine, and thymidylate [98]. Furthermore, it plays a critical role in the maintenance of NAD+/NADH and NADP+/NADPH ratios: NADH facilitates mitochondrial oxidative phosphorylation and ATP production, while NADPH provides the reducing equivalents that are necessary for antioxidant defense, such as the reduction of glutathione disulfide (GSSG) to glutathione (GSH) [99].

Glycine further supports one-carbon metabolism by supplying one-carbon units to the folate and methionine cycles, and the trans-sulfuration pathway, facilitating the biosynthesis of porphyrins, purines, and S-adenosylmethionine (SAM) [103,104]. Beyond its role in redox homeostasis and epigenetic regulation, glycine is also required for the synthesis of cysteine- and glycine-rich protein 2, which promotes metastasis and chemoresistance via Notch pathway activation [105,106].

Additionally, serine also functions as an allosteric activator of Pyruvate Kinase M2 (PKM2), a key regulator of the glycolytic flux, thereby sustaining aerobic glycolysis and influencing pyruvate fate toward lactate production or entry into the TCA cycle. Thus, a metabolic feedback loop exists in which serine also stimulates glycolytic activity, thereby reinforcing the glycolytic metabolic flux and promoting tumor growth. Through this integration, the ser/gly pathway operates not merely as a branch of glycolysis but as a metabolic circuit that coordinates energy production with anabolic demand [99].

### Advances in GBM Treatment: Current Strategies and Future Perspectives in Targeting the Ser/Gly Metabolic Axis

Existing GBM therapies offer limited survival benefit due to intratumoral heterogeneity, tumor recurrence, and intrinsic resistance mechanisms, highlighting the critical need for innovative treatment approaches [107]. The standard of care is maximal safe surgical resection supported by imaging technologies to minimize the risk of neurological deficits [108,109,110,111,112]. Temozolomide (TMZ) is the first-line chemotherapeutic agent; however, its efficacy is constrained by DNA repair mechanisms and diverse signaling pathways that contribute to chemoresistance [113,114,115]. In younger patients, the concurrent administration of radiotherapy with adjuvant TMZ remains the gold standard, significantly improving 2–5-year survival outcomes, even though it demonstrates limited efficacy in recurrent gliomas [116].

Recent therapeutic advances involving immunotherapies have shown efficacy in preclinical models, but the available clinical data in patients with GBM remain limited and not yet encouraging [117,118,119,120,121,122,123,124,125], while glycolysis inhibitors, HIF-targeting agents, and immune checkpoint therapies have been associated with substantial toxicity [126]. Other novel approaches selectively disrupt signaling pathways for tumor growth while minimizing off-target toxicity [107,127,128].

As ser/gly pathway hyperactivation is a hallmark of cancer, Sánchez-Castillo et al. explored the use of repurposed drugs with established blood–brain barrier (BBB) permeability, such as Sertraline, a common antidepressant, and Chloroquine, an anti-malaria drug. The goal was to selectively target the ser/gly biosynthetic pathway, which is upregulated in approximately 30% of cancers and hyperactivated in ~25% of GBM cases [99]. Their in vitro approach focused on murine and patient-derived GBM cells, characterized by the hyperactivation of the ser/gly pathway, often driven by PSPH amplification, which also promotes the expression of the immunosuppressive protein galectin-1.

In the proposed therapeutic strategy, Sertraline inhibits SHMT1/2, thereby reducing ser/gly synthesis, impairing mitochondrial function, and suppressing galectin-1 expression. Concurrently, Chloroquine compromises mitochondrial integrity by depolarizing the mitochondrial membrane and inhibiting autophagic clearance of dysfunctional mitochondria, resulting in organelle accumulation and enhanced cellular stress. The combination of Sertraline and Chloroquine thus exerts a synergistic, selective inhibition of proliferation in ser/gly-high GBM cells, reducing cell survival by up to 60% in sensitive models.

Mechanistically, the suppression of the ser/gly pathway impairs nucleotide synthesis, depletes NADH and ATP, reduces TCA cycle intermediates and aspartate, and induces mitochondrial morphological defects. Notably, the efficacy of this combination is enhanced under hypoxic and low-glucose conditions, which are fundamental characteristics of the GBM microenvironment, highlighting its potential as a targeted therapy for the ser/gly-high subset and as a strategy to improve immunotherapeutic responses [99].

Within the framework of cancer metabolic reprogramming, PHGDH has emerged as the key rate-limiting enzyme of the de novo serine synthesis pathway [129]. Recent evidence has demonstrated a marked upregulation of PHGDH in GBM cells, with particularly high expression in glioma stem-like cells (GSCs). Functionally, PHGDH enhances serine production and fuels downstream one-carbon metabolism, leading to increased NADPH generation. This supports GSH- and thioredoxin-dependent antioxidant systems, thereby maintaining redox homeostasis. By limiting the accumulation of reactive oxygen species (ROS), this metabolic program preserves GSC self-renewal capacity, sustains the expression of stemness-associated markers such as SOX2 and Olig2, and promotes efficient DNA repair [130].

The therapeutic relevance of this pathway has been investigated through both pharmacological and genetic inhibition of PHGDH. Pharmacological targeting was achieved using the BBB-permeable inhibitor NCT-503, while genetic suppression was implemented through CRISPR–Cas9, shRNA, and inducible knockdown approaches. Across experimental models, PHGDH inhibition consistently reduced GSC self-renewal, downregulated stemness markers, and enhanced radiosensitivity in both GBM cells and GSCs, in vitro and in vivo. Comparable effects were also observed following dietary restriction of serine and glycine, further supporting the potential of PHGDH targeting as a strategy to overcome radioresistance and improve therapeutic outcomes in GBM (Figure 3) [130].

Beyond its role in GBM cells, PHGDH has emerged as an important regulator of tumor vasculature. Its expression is selectively increased in tumor-associated endothelial cells compared with normal brain endothelium, largely driven by ATF4 activation in response to microenvironmental stressors such as chronic hypoxia and VEGF-A-mediated pro-angiogenic signaling. This metabolic reprogramming promotes abnormal endothelial proliferation and aberrant vessel sprouting, contributing to the disorganized vascular architecture, typical of GBM microenvironment [131].

Genetic ablation of PHGDH in endothelial cells reduces excessive vascular sprouting, alleviates intratumoral hypoxia, and enhances T-cell infiltration within tumors. PHGDH inhibition consequently promotes antitumor T-cell immunity and increases GBM sensitivity to CAR T-cell therapy. These results suggest that targeting PHGDH to reprogram endothelial metabolism may represent a promising strategy to improve the efficacy of T-cell–based immunotherapies [131].

Collectively, these findings suggest that PHGDH inhibition may exert multifaceted therapeutic effects by simultaneously disrupting ser/gly metabolism while also suppressing tumor-associated angiogenesis and aberrant vasculature formation, thereby limiting both the metabolic and structural support required for GBM progression.

In addition to Sertraline, Chloroquine, and PHGDH inhibition, the methionine and folate cycle represents another pathway of particular interest. Indeed, short-term inhibition of the methionine cycle or pharmacological targeting of MAT2A has been shown to impair the tumor-initiating capacity of GBM tumorsphere cells. This finding is particularly relevant in the context of hypoxia-driven metabolic rewiring, which promotes the downregulation of DHFR, a key enzyme in one-carbon metabolism, alongside the concomitant upregulation of MAT2A in GBM tumorspheres, thereby enhancing their proliferative capacity independently of exogenous folate availability [132]. Collectively, these observations highlight additional and promising pharmacological targets for GBM; however, their therapeutic potential has so far been demonstrated exclusively in vitro, warranting further validation in more physiologically relevant preclinical models. Importantly, the metabolic landscape of GBM cells is highly complex and deeply integrated with multiple adaptive and compensatory pathways, which must be carefully considered when evaluating the feasibility and robustness of metabolic targeting strategies.

## 6. Conclusions, Limitations, and Future Perspective

In this review, we have journeyed through the complex landscape of gut dysbiosis, exploring how microbial alterations shape ser/gly metabolism. Using insights from murine models, we highlighted the gut’s influence on brain functions. We further connected these microbial-driven metabolic pathways to hepatic processes, emphasizing the liver’s role in producing secondary metabolites such as taurine and tauroconjugated biliary salts that potentially impact gut and brain homeostasis. Emerging evidence increasingly highlights a compelling link between gut dysbiosis and the development of diseases, with GBM standing out as a striking example of how microbial imbalances may influence tumor onset and progression.

We summarized evidence on how gut microbiota alteration may be considered as a contributing factor to GBM progression, particularly through the accumulation of glycine, reported to increase under dysbiotic conditions and implicated in promoting tumor growth. Consistently, elevated glycine concentrations have also been observed in the peritumoral area of GBM patients, supporting its role in tumor metabolism and aggressiveness, and linking GBM pathophysiology to the ser/gly biosynthetic axis.

It may therefore be postulated that these two processes reciprocally potentiate one another; however, further investigations are required to elucidate more precisely the temporal sequence and mechanistic relevance of the predisposing and/or causative events underlying tumor progression.

As current therapies, including surgery, radiotherapy, chemotherapy, and immunotherapy, remain constrained by intrinsic resistance such as intratumoral heterogeneity, and limited blood–brain barrier penetration, there has been growing interest in targeting the ser/gly axis. Notably, repurposed agents such as Sertraline and Chloroquine, which readily cross the BBB, have been shown to selectively disrupt ser/gly metabolism, impairing mitochondrial function and tumor growth; however, quantitative data specifically defining the brain concentrations attainable in humans at clinically tolerated doses remain unavailable.

Through this narrative, we have synthesized current evidence to highlight the pivotal role of the gut microbiota in glycine metabolism and its function as a central mediator along the gut–liver–brain axis, which still requires further integration in the GBM context to achieve a clearer understanding of this inter-organ communication, a goal that—while already challenging to address in animal disease modeling—becomes even more difficult to attain in clinical studies.

Commonly administered supportive therapies in glioblastoma patients, including dexamethasone, antiepileptic drugs, antibiotics, and dietary modifications, may represent major confounding factors in GBM cohorts, as they can profoundly influence immune responses, tumor metabolism, treatment sensitivity, and gut microbiota composition, thereby complicating the interpretation of clinical and translational findings. In addition, the precise molecular mechanisms linking microbial metabolites to metabolic reprogramming in GBM remain largely unresolved. Collectively, these limitations also open important avenues for integrative, longitudinal, and mechanistic studies aimed at disentangling the complex crosstalk among the gut microbiota, tumor biology, and metabolic rewiring in glioblastoma.

**Figure 4:**
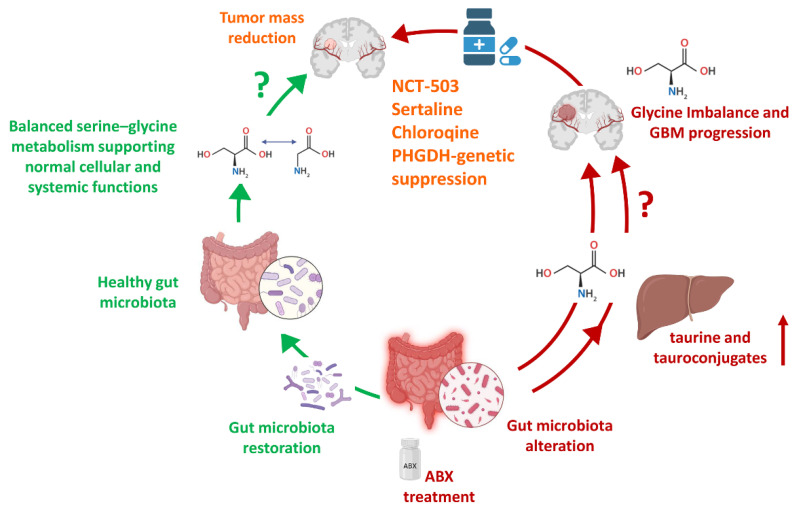
**Schematic overview linking gut dysbiosis, liver, Ser/Gly metabolism, and GBM.** Induced by antibiotic administration, alterations of the gut microbiota can lead to dysregulation of ser/gly metabolism, resulting in increased production of taurine and tauroconjugates. This metabolic imbalance has been associated with enhanced tumor progression and poorer prognosis in GBM. In addition, microbiota-driven changes in glycine metabolism can affect liver function, promoting the production of tauroconjugates and bile salts. Targeting this pathway represents a promising therapeutic strategy, as it is essential for tumor cell proliferation. Compounds such as NCT-503, Sertraline, and Chloroquine, as well as genetic suppression of PHGDH, may represent alternative approaches to inhibit glioma growth by interfering with this critical metabolic pathway. Restoration of a healthy gut microbiota may also support normal brain metabolic homeostasis by maintaining physiological glycine metabolism. The red arrows represent the pathological context, while the green arrows indicate the potential physiological context supporting a proper glycine/serine balance.

Looking forward, the ser/gly axis emerges as a central metabolic vulnerability in GBM, and its therapeutic targeting—potentially in combination with strategies to modulate dysbiosis—represents a promising precision approach to constrain tumor progression (Figure 4).

## Data Availability

Not applicable.

## Abbreviations

The following abbreviations are used in this manuscript:

ABX	antibiotic
CNS	central nervous system
CSF	cerebrospinal fluid
ENS	enteric nervous system
GALT	gut-associated lymphoid tissue
HP axis	Hypothalamic-Pituitary-Adrenal axis
CRH	Corticotropin-Releasing Hormone
ACTH	Adrenocorticotropic Hormone
GBM	Glioblastoma multiforme
GF	germ-free
GSC	glioma stem cell
IDH	isocitrate dehydrogenase
NMR	nuclear magnetic resonance
3PG	3-phosphoglycerate
PHGDH	phosphoglycerate dehydrogenase
3-PHP	3-phosphohydroxypyruvate
PSAT1	phosphoserine aminotransferase 1
3-PS	3-phosphoserine
PSPH	phosphoserine phosphatase
PKM2	Pyruvate Kinase M2
dTMP	thymidylate
SAM	S-adenosylmethionine
α-KG	α-ketoglutarate
GSSG	Glutathione disulfide
GSH	Glutathione
ser/gly	serine/glycine
SHMT1/2	serine hydroxymethyltransferase 1/2
TCA	tricarboxylic acid
TMZ	temozolomide
BBB	blood–brain barrier
